# Incidence of subchorionic hematoma and contributing factors in assisted reproductive technologies—a retrospective cohort study

**DOI:** 10.3389/fmed.2025.1569789

**Published:** 2025-04-23

**Authors:** Wei Wei, Xue Chang Qiu, Ni Tang, Zhuo Liang, Jinxiang Wu, Pinxiu Huang

**Affiliations:** ^1^Center of Reproductive Medicine, Guangzhou Women and Children’s Medical Center-Liuzhou Hospital, Liuzhou, Guangxi, China; ^2^Center of Reproductive Medicine, The First Affiliated Hospital of Guilin Medical University, Guilin, Guangxi, China; ^3^Department of Reproductive Medicine, The Second Affiliated Hospital of Fujian Medical University, Quanzhou, Fujian, China

**Keywords:** subchorionic hematoma, *In vitro* fertilization, preimplantation genetic testing, frozen–thawed embryo transfer, miscarriage, preterm birth

## Abstract

**Background:**

To explore the incidence of subchorionic hematoma (SCH) in IVF-ET (*In vitro* fertilization-embryo transfer, IVF-ET) fresh, IVF-FET (*In vitro* fertilization-freeze–thaw embryo transfer, IVF-FET), PGT-FET (preimplantation genetic testing-freeze–thaw embryo transfer, PGT-FET), AIH (artificial insemination by husband, AIH), and natural pregnancy (NP), and to analyze contributing factors.

**Methods:**

This is a retrospective cohort study. Patients were included: IVF-fresh ET, IVF-FET, PGT-FET, AIH, and NP patient groups. The incidence of SCH in different ART and effect of SCH on pregnancy outcome were compared, Further, multivariate analyses of the occurrence of SCH were conducted.

**Results:**

The incidence of SCH with IVF-fresh ET, IVF-FET, PGT-FET, AIH and NP was 27.50%, 22.56%, 16.86%, 12.95%, and 13.02%, respectively. Compared with the incidences of SCH with AIH and NP that for IVF-fresh ET and IVF-FET transfer were significantly increased (*p* < 0.05). The occurrence of SCH was not significantly associated with miscarriage and was significantly negatively correlated (*p* < 0.05) with high-quality embryos.

**Conclusion:**

The incidence of SCH in ET was increased compared with that in the Not ET groups, especially after fresh ET. ET unavoidably seems to contribute to the development of SCH; however, it does not affect the pregnancy outcome.

## 1 Introduction

Subchorionic hematoma (SCH) refers to bleeding caused by a separation between the chorionic plate and the bottom decidua that makes blood accumulate between the villus and the bottom decidua. Ultrasound imaging shows a dark liquid area between the uterine wall of the uterine cavity and the pregnancy sac. The incidence of SCH varies from 0.46% to 39.5% in the obstetric population (1). In recent years, with continuous advancements in assisted reproductive technology (ART), infertile couples have increasingly availed of in IVF-ET treatments, and SCH events have become increasingly common. The etiology of SCH and whether it produces adverse pregnancy outcomes are currently unclear. First-trimester SCH reportedly has a close relationship with poor pregnancy outcomes such as pregnancy loss, preterm delivery, and abruption (2–4). However, some studies have found no association between SCH and adverse perinatal outcomes (1). Some studies have shown that SCH is more likely in IVF patients than in non-IVF patients (5). Further, some studies have reported that SCH is less likely with thawed ET (6), whereas others have contrastingly reported that it is less likely with fresh ET (5). Therefore, the incidence of SCH with different human ARTs remains unclear.

Currently available ARTs include natural pregnancy, AIH, IVF, intracytoplasmic sperm injection (ICSI), frozen–thawed embryo transfer (FET), and preimplantation genetic testing (PGT). However, few studies have explored the incidence of SCH and its impact on pregnancy outcomes after the use of different ARTs. The etiology of SCH is complex, resulting in unmanaged patients or excessive treatment. Therefore, we further clarified the incidence of SCH and analyzed contributing factors with the use of the abovementioned ARTs for clinical reference.

## 2 Object and method

### 2.1 Research object

**Figure 1:**
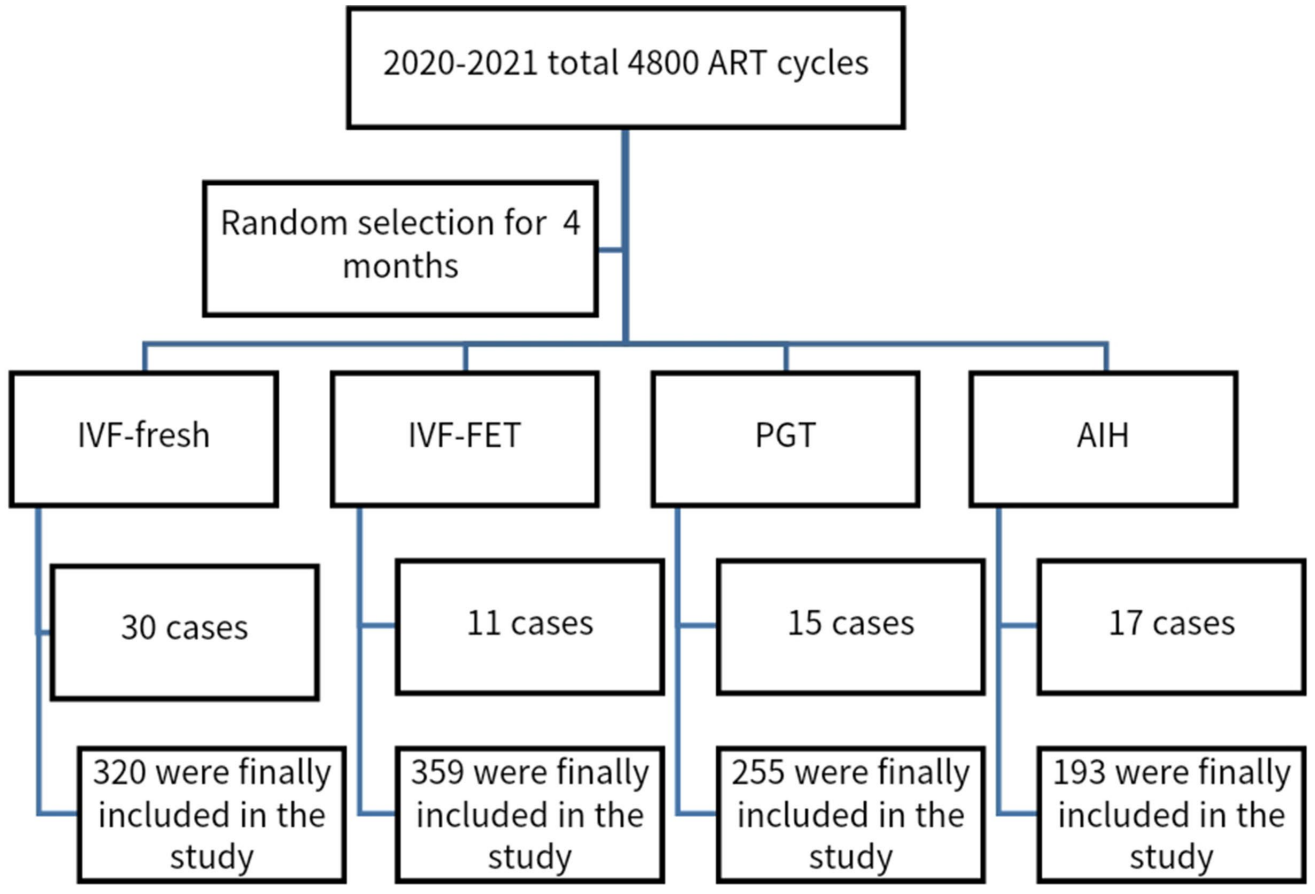
Flow chart: Data from the 4 months in the same period of different ART pregnancy aid schemes in 2020–2021 were randomly selected.

We used a retrospective cohort study method to randomly select a 4-month period in 2020–2021, during which a population at the Reproductive Medicine Center of Guangzhou women and children medical treatment center Liuzhou hospital was chosen, as shown in Figure 1. This study was approved by the ethics committee of Guangzhou women and children medical treatment center Liuzhou hospital. Due to the retrospective nature of the study, the informed consent was waived by the ethics committee of Guangzhou women and children medical treatment center Liuzhou hospital. All methods were performed in accordance with the relevant guidelines. SCH cases were not systematically recorded in our ART pregnancy outcome database. To include SCH in our analysis, we manually extracted and reviewed the records. Given these limitations, we selected a four-month dataset for evaluation.

The inclusion criteria were the presence of an intrauterine single living fetus between 25 and 42 days after ET, and single fetal heartbeat appearing on ultrasound at 35 and 42 days after ET or AIH. In natural pregnancy, it is 7–10 weeks.

Exclusion criteria were also applied, which were: (1) Patients diagnosed with exceptional immunity, including antiphospholipid syndrome; (2) Patients diagnosed with anatomical abnormalities of the genital tract through gynecological ultrasound, salpingography, or laparoscopy; (3) Patients who had maternal diseases such as diabetes, hypertension; (4) Patients diagnosed with endocrine abnormalities; (5) Patients with prethrombotic conditions; (6) Patients who had reproductive tract infections. Those who met the inclusion criteria were classified into IVF-fresh transplant (*n* = 350), IVF-FET (*n* = 370), PGT-FET (*n* = 270), and AIH (*n* = 210). During the follow-up, some patients were found to have had B-ultrasound examination at 35 and 42 days in other hospitals; however, telephone follow-ups were insufficient to determine the presence of SCH. Therefore, these members of the population were lost to follow-up. Finally, individuals with IVF-fresh transplant (*n* = 320), IVF-FET (*n* = 359), PGT-FET (*n* = 255), and AIH (*n* = 193) were included in the study. we added the incidence of SCH in natural pregnancy. In addition, we chose the incidence of SCH in natural pregnancy (*n* = 215) that meets the criteria in the same period as a comparison. The management for all pregnancies complicated by SCH was to recommend bed rest.

### 2.2 Methods

#### 2.2.1 Fresh ET

As a gonadotropin-releasing hormone (GnRH) agonist long scheme, GnRH-a (1.875 mg/d) (Triptorelin Acetate, Ipsen France Biotechnology Company) was used in the previous middle luteal phase. After 20 days, according to the patient age, number of antral follicles, basic hormones, and previous ovarian response, 75–300 IU recombinant follicle-stimulating hormone (rFSH, Gonafen, Serrano Company, Switzerland) was initially administered. Subsequently, the gonadotrophin (Gn) dosage was adjusted according to the follicular size and hormone changes.

For the GnRH antagonist scheme, rFSH was used from the 2nd or 3rd day of menstruation. According to the patient age, number of basal antral follicles, and basic hormones, 75–300 IU rFSH was initially administered. Subsequently, the rFSH dosage was adjusted according to the follicle size and hormone changes. GnRH-ant (Sizekai, Serrano, Switzerland) was used when the diameter of the follicles was 12–16 mm, and 0.25 mg of Sizekai was injected intramuscularly every day until the injection date of human chorionic gonadotropin (hCG).

The trigger time of hCG (Zhuhai Lizhu Company, China) injection was as follows: when 2–3 follicles had a diameter of ≥18 mm, hCG was injected intramuscularly to induce ovulation, and after 34–36 h, transvaginal ultrasound-guided oocyte retrieval was performed. According to the situation, conventional IVF or ICSI was used for fertilization. ET was conducted under ultrasound guidance at 3–5 days after oocyte retrieval.

#### 2.2.2 Thawed ET

In the natural cycle, follicles were monitored on day 10 of menstruation. When the average diameter of the follicles was 16 mm, luteinizing hormone (LH), estradiol (E2), and progesterone (P) levels were measured. When blood E2 > 549 pmol/L and LH peaked, 6,000 IU of hCG was injected. On day 2 after hCG injection, oral dydrogesterone tablets (10 mg/Tid) and hCG 2,000 IU/Q3d injection were given. Finally, frozen ET was performed after 3–5 days.

In the artificial cycle, estradiol valerate 2 mg/Bid was started for 7 days from day 2–4 of menstruation, and then, a dose of 3 mg/Bid was given for another 7 days. When the endometrial thickness was ≥8 mm, dydrogesterone tablets (10 mg/Tid) and progesterone soft capsules (200 mg/Tid) were given to transform the inner membrane. Finally, ET was performed 3–5 days after transformation.

#### 2.2.3 Embryonic freezing method

All embryos were frozen using the vitrification cryopreservation method. Frozen embryos were thawed rapidly on the morning of the day of transplantation, as described in reference (7).

#### 2.2.4 PGT-FET

When both parents are thalassemia gene carriers, there is a 25% chance that their offspring will have severe thalassemia. Patients can choose PGT for monogenic disorders (PGT-M) + PGT for aneuploidies (PGT-A). Patients who have balanced rearrangements or Roche translocation can chose PGT for chromosomal structural rearrangements (PGT-SR). Patients with recurrent abortion or repeated implant failure can chose PGT-A. After the antagonist protocol, conventional embryos were cultured for inspection. After preparing for the endometrium in the natural or artificial cycle, the available embryos were thawed and transplanted.

#### 2.2.5 AIH

During normal menstrual rules, vaginal B-ultrasound monitors were used to observe follicle development on the 10th day of menstruation. Patients with ovulation disorders can use an ovulation induction program, in which letrozole or letrozole + HMG is begun 3–5 days after menstruation. Follicular development was monitored through vaginal B-ultrasound, and when the follicle diameter reached a maximum of 18 mm, 5,000–10,000 IU of hCG was injected intramuscularly, and intrauterine insemination was performed 48 h after intramuscular injection.

#### 2.2.6 Observational indicators

The incidence of SCH, abortion rate, prematurity rate, and term incidence rate were evaluated. B-ultrasound examination by experienced doctors at 7–10 weeks pregnant revealed the uterine cavity, crescent between the gestational sac, and triangular or irregular anechogenic areas, which are indicative of SCH. Gestational sacs as revealed by ultrasonography were defined as clinical pregnancy. A miscarriage occurring before 12 weeks of gestation is defined as a miscarriage, delivery occurring before 37 weeks of gestation is defined as a premature delivery, and delivery after at least 37 weeks of gestation is defined as a full-term delivery. Prevalence of SCH = number of SCH cycles/clinical pregnancy cycles × 100%. Abortion rate = number of abortion cycles/number of clinical pregnancy cycles×100%. Prematurity rate = number of preterm cycles/clinical pregnancy cycles × 100%. Incidence of term = Number of term birth cycles/clinical pregnancy cycles×100%.

#### 2.2.7 Statistical analysis

Statistical processing was performed using SPSS 13.0 software. Measurement data were expressed as X ± S, and group means above three groups were analyzed by ANOVA. Percentage comparisons were performed using the chi-square test, and binary logistic regression was used to analyze which factors were associated with the occurrence of SCH. *p* < 0.05 was considered statistically significant.

## 3 Results

This study involved 1,342 patients including IVF-fresh transplant (*n* = 320), IVF-FET (*n* = 359), PGT-FET (*n* = 255), and AIH (*n* = 193), and natural pregnancy (*n* = 215) (Figure 1).

The mean age of the five groups was similar, and their incidences of SCH were 27.50%, 22.56%, 16.86%, 12.95%, and 13.02%, respectively. There incidence of SCH was the highest in the IVF-fresh ET and lowest in the AIH and NP groups, respectively. After IVF, the incidence of SCH in the fresh embryo group (27.50%) and FET group (22.56%) was significantly higher than that in the AIH group (12.95%) and the NP group (13.02%), with *p* < 0.05. Further, the incidence of SCH in the IVF-fresh ET group (27.50%) was significantly higher than that in the PGT-FET group (16.86%), with *p* < 0.05. The IVF fresh ET and IVF-FET groups did not show a significant difference in the incidence of SCH (27.50% vs. 22.56%). The incidence of SCH in the AIH group and the NP group was similar. The incidences of SCH in the ET blastocyst and in the ET embryo was similar (Table 1).

**Table 1:** The incidence of subchorionic hematoma under different ATR pregnancy-aid regimens.

Group	IVF-fresh ET	IVF-FET	PGT-FET	AIH	Non ART	*p* value
Number of people	320	359	255	193	215	
Age (year)	33.15 ± 6.52	33.37 ± 6.45	32.95 ± 7.08	32.76 ± 8.34	33.23 ± 7.89	>0.05
Incidence of SCH %	27.50% (88/320)^a^	22.56% (81/359)^b^	16.86%^#^ (43/255)	12.95%^*^ (25/193)	12.56%^*^ (27/215)	<0.05
The incidence of SCH in the ET blastocysts%	28.10% (43/153)	22.77% (69/303)	NA	NA		>0.05
The incidence of SCH in ET embryos%	26.95% (45/16)	21.42% (12/56)	NA	NA		>0.05

^*^Significant decrease as compared to a and b (AIH compared to IVF-fresh ET and IVF-FET *p* < 0.05). ^*^Significant decrease as compared to a and b (Non ART compared to IVF-fresh ET and IVF-FET, *p* < 0.05). ^#^Significant decrease as compared to a (PGT-FET compared to IVF-fresh ET *p* < 0.05). IVF-fresh ET and IVF-FET were similar. IVF-FET and PGT-FET were similar. AIH, Non ART, and PGT-FET were similar.

In the different ARTs group, no significant differences were seen between the SCH and non-SCH groups in terms of the rates of abortion, preterm birth, and term birth, *p* > 0.05. Therefore, the occurrence of SCH does not affect the outcome of pregnancy with different ART methods in that it does not have a significant association with the abortion, preterm birth, and term birth rates (Table 2).

**Table 2:** Effect of subchorionic hematoma on pregnancy outcome.

Group	IVF-fresh ET	IVF-FET	PGT-FET	AIH
Subchorionic hematoma abortion ratio%	9.09% (8/88)	13.58% (11/81)	2.32% (1/43)	4.00% (1/25)
No subchorionic hematoma abortion ratio%	9.05% (21/232)	7.91% (22/278)	2.83% (6/212)	11.31% (19/168)
*p* value	>0.05	>0.05	>0.05	>0.05
Subchorionic hematoma preterm birth rate%	3.41% (3/88)	6.17% (5/81)	2.33% (1/43)	8.00% (2/25)
No subchorionic hematoma preterm birth rate%	9.05% (21/232)	5.40% (15/278)	2.36% (5/212)	7.74% (13/168)
*p* value	>0.05	>0.05	>0.05	>0.05
Subchorionic hematoma incidence of term births %	84.09% (74/88)	76.54% (62/81)	93.02% (40/43)	88.00% (22/25)
No subchorionic hematoma incidence of term births%	81.03% (188/232)	84.89% (236/278)	94.34% (200/212)	79.76% (134/168)
*p* value	>0.05	>0.05	>0.05	>0.05

A total of 320 individuals underwent fresh ET, following which 88 developed SCH and 232 did not. The SCH and non-SCH patient groups did not show statistically significant differences in age, foundation FSH, hCG day endometrial thickness, hCG day E2 level, and proportion of transplanted blastocysts, indicating that SCH was not associated with these parameters (Table 3).

**Table 3:** Analysis of causes with or without subchorionic hematoma in fresh embryo transfer.

Group	With SCH	Without SCH	*p* value
Number of people	88	232	
Age (y)	33.17 ± 4.38	33.14 ± 4.49	>0.05
Foundation FSH	5.64 ± 1.73	5.85 ± 1.90	>0.05
HCG day endometrial thickness	12.03 ± 3.11	11.38 ± 2.81	>0.05
HCG day E2 level	1902.67 ± 1040.71	1881.16 ± 1015.81	>0.05
The proportion of transplanted blastocysts%	47.72% (42/88)	48.89% (110/225)	>0.05

In IVF-FET with 239 natural cycles and 120 artificial cycles, no statistically significant difference in age was seen. Further, the incidence of SCH was 19.67% and 17.50%, respectively, with no statistically significant difference (Table 4).

**Table 4:** Incidence of subchorionic hematoma in the natural vs. artificial cycles of IVF-FET.

Group	Artificial cycles	Natural cycles	*p* value
Number of people	120	239	
Age (y)	33.41 ± 5.35	34.27 ± 4.56	>0.05
SCH incidence	17.50 (21/120)	19.67 (47/239)	>0.05

The incidence of SCH under PGT-A, PGT-SR, and PGT-M + A was 14.04, 13.46, and 19.18%, respectively, with no statistically significant difference (Table 5).

**Table 5:** Incidence of subchorionic hematoma under different PGT pregnancy-aid regimens.

Group	PGT-A	PGT-SR	PGT-M + A	*p* value
Number of people	57	52	146	
SCH incidence	14.04 (8/57)	13.46 (7/52)	19.18 (28/146)	>0.05

Age, foundation FSH, intimal thickness, ET count, embryo quality, presence of blastocysts, and hCG day E2 level were included in the fresh ET group to construct the binary logistic regression equation. The incidence of SCH was found to be significantly negatively correlated with high-quality embryos (B = −1.139), with *p* < 0.05. However, the incidence of SCH was not significantly associated with age, foundation FSH, intimal thickness, ET count, presence of blastocysts, and hCG day E2 level (Table 6).

**Table 6:** Binary regression analysis of the subchorionic hematoma.

	Significance	EXP (B)	The 95% confidence interval for the EXP (B)	
			Lower limit	Upper limit
Age	0.686	0.987	0.929	1.050
Foundation FSH	0.516	0.952	0.821	1.104
Intimal thickness	0.280	1.055	0.957	1.162
ET count	0.481	1.436	0.525	3.928
Whether high-quality embryos	0.022	0.320	0.120	0.850
Whether blastocyst	0.597	1.309	0.483	3.548
HCG day E2 level	0.953	1.000	1.000	1.000
Constant	−1.456	0.384	0.233	

## 4 Discussion

The etiology of and risk factors for the incidence of SCH remain unclear. We studied the incidence of SCH with AIH, IVF, FET, and PGT treatments and found that the incidence of SCH after ET was higher than that of SCH after AIH. In order to be more convincing, we added the incidence of SCH in natural pregnancy. Surprisingly, the incidence of SCH in the AIH group and the NP group was similar and also lower than that in the ET cycle. In short, the incidence of SCH in ET was increased compared with that in the Not ET groups.

We believe that the SCH may be due to the unavoidable invasion of blood vessels after embryo implantation into the uterine cavity. The incidence of SCH was low after non-ET because of the mutual interaction between the endometrium and zygote that from fertilization to cleavage to blastocyst formation. These signals make the embryo adhesion and invasion process more gentles, and the invasion process has an appropriate protective effect on the endometrial blood vessels. The incidence of SCH was the highest after fresh ET because the excessive physiological dose of estrogen increases the thickness of the endometrium, promotes vascular formation, and increases the permeability of blood vessels, making embryo invasion into the endometrium more likely to invade blood vessels, leading to vascular rupture and bleeding. Therefore, the incidence of SCH after fresh ET is higher than that after FET. However, the incidence of SCH after PGT-FET was significantly lower than that after IVF fresh ET. To investigate this further, we performed a binary regression analysis of the factors associated with SCH in patients with fresh ET and found that the transfer of high-quality embryos was negatively associated with the incidence of SCH. Moreover, our PGT patients underwent PGT-A testing, which excluded chromosome aneuploidy. These embryos were of high quality, and after blastocyst biopsy, zona pellucida damage, and possibly decreased blastocyst invasive ability, the incidence of SCH after PGT-FET was low. The increase in the incidence of SCH after IVF fresh ET and after IVF-FET may also be cause by unknown factors affecting infertility. For example, the incidence of SCH has been attributed to immunity and inflammation (8).

Similar to the present study, Zhou retrospectively analyzed 1,097 patients who were successfully conceived by IVF embryo transfer (IVF-ET) within a certain 6-month period and found that the incidence of SCH was 12.1% (133/1,097). Moreover, the incidence of SCH in the fresh ET group was higher than that in the freeze–thaw ET group, suggesting that fresh ET increased the risk of SCH, possibly owing to ovarian stimulation and high estradiol levels in patients (6). Ma compared 390 fresh ET and 392 frozen ET patients and found a higher incidence of SCH in the former than in the latter (36.41% vs. 29.08%). Further, the incidence increased with the number of eggs produced, possibly owing to the higher estrogen level in such patients interfering with the function of endometrial blood vessels (9). Some studies believe that the higher the level of embryonic trophectoderm, the lower is the incidence of SCH (10). This is consistent with the conclusion that the incidence of SCH after PGT-FET decreased significantly compared with that after fresh ET owing to high-quality embryos.

In contrast to the present study, Kozue concluded that the incidence of SCH is higher with frozen–thawed embryos than with fresh ET. This may be because all frozen ETs in this study underwent hormone replacement therapy (HRT) and exogenous estrogen stimulation of the ovary, leading to abnormal placenta implantation and vascular rupture and bleeding when the villus invaded the endometrium (5). In another study, the use of HRT in FET increased the incidence of SCH relative to that in the natural cycle (11). In that study, the E2 level of the HRT regimen was higher than that in the natural cycle; however, further analysis showed that the estradiol level did not affect the incidence of SCH. However, this study suggest that the incidence of SCH in natural cycle FET was similar to that of SCH in the HRT regimen (19.67% vs.17.50%), and the difference was not statistically significant (*p* > 0.05). Ma’s conclusion was similar to ours, in that during the freeze–thaw ET cycle, the incidence of SCH with HRT was not significantly different from that with the natural cycle (30.26% vs. 27.43%, *p* = 0.544) (9).

At present, the clinical significance of SCH is unclear, however, the increasing incidence of SCH after IVF-ET is raising questions over whether SCH affects pregnancy outcomes (6, 11). Ma concluded that SCH does not result in statistically significant differences in neonatal weight, premature birth rate, and delivery mode; however, SCH patients had a higher incidence of miscarriage (25.40% vs. 13.46%, *p* = 0.035) (9). West noted no difference in spontaneous abortion rates between IVF pregnancies with and without SCH (18.5% (12/65) vs. 15.2% (22/145), *p* = 0.69) (10). Zhou divided 1,097 fresh/thawed ET cycles into SCH and non-SCH groups and found no significant differences in early pregnancy abortion rate, fetal loss rate, live birth rate, or birth defect rate; however, the birth weight in the SCH group was significantly lower than that in the non-SCH group (3207.8 ± 595.7 g vs. 3349.2 ± 559.7 g, *p* = 0.03) (6). Eaton also reported no statistically significant correlation between early vaginal bleeding and live birth rate in twins who underwent IVF; however, they found that the birth weight in twin IVF with SCH was lower than that without SCH (12). In contrast, Xiang found no association between SCH and birth weight; however, they found that the rate of prematurity was higher in patients with SCH than in those without SCH (13). The results of this study are consistent with those of the retrospective 2019 cohort study by Naert, in which univariate analysis of 2,446 pregnant women revealed that SCH was not associated with adverse pregnancy outcomes over 20 weeks of gestation (including preterm birth, neonatal low body weight, placental abruption, or intrauterine fetal death over 20 weeks of gestation), and hematoma size was not associated with pregnancy outcome (1). This study found that the abortion, premature birth, and term birth rates were not significantly different among different ART methods with and without SCH. We believe that the occurrence of SCH does not affect pregnancy outcomes and does not require excessive intervention.

This study concluded that ET contributes to the development of SCH, and this seems a normal phenomenon. The SCH area is mostly smaller than the pregnancy sac, and therefore, it does not affect the pregnancy outcome and does not require intervention. The innovative point of our study is the inclusion of PGT-FET data in the analysis, revealing that the incidence of SCH after PGT-FET was significantly reduced compared with that after fresh ET. Moreover, the incidence of SCH after FET with PGT-A, PGT-M + A, and PGT-SR methods is similar, indicating that high-quality embryos and blastocyst biopsy both lead to a decrease in SCH.

The findings of this study are limited by its retrospective design. Further, we were unable to obtain data on any potential confounding factors, such as immunological abnormalities, smoking, previous obstetric complications, and family history. Studies with larger sample sizes are needed to determine the risk factors for SCH in IVF pregnancies.

## Data Availability

The raw data supporting the conclusions of this article will be made available by the authors, without undue reservation.
